# Nitrative Stress and Tau Accumulation in Amyotrophic Lateral Sclerosis/Parkinsonism-Dementia Complex (ALS/PDC) in the Kii Peninsula, Japan

**DOI:** 10.3389/fnins.2017.00751

**Published:** 2018-01-22

**Authors:** Yukiko Hata, Ning Ma, Misao Yoneda, Satoru Morimoto, Hideyuki Okano, Shigeo Murayama, Shosuke Kawanishi, Shigeki Kuzuhara, Yasumasa Kokubo

**Affiliations:** ^1^Department of Neurology, Graduate School of Medicine, Mie University, Mie, Japan; ^2^Division of Health Science, Graduate School of Health Science, Suzuka University of Medical Science, Mie, Japan; ^3^Department of Medical Welfare, Suzuka University of Medical Science, Mie, Japan; ^4^Department of Oncologic Pathology, Graduate School of Medicine, Mie University, Mie, Japan; ^5^Department of Physiology, Keio University School of Medicine, Tokyo, Japan; ^6^Department of Neuropathology, Metropolitan Geriatric Hospital and Institute of Gerontology, Tokyo, Japan; ^7^Faculty of Pharmaceutical Sciences, Suzuka University of Medical Science, Mie, Japan; ^8^Department of Neurology and Medicine, School of Nursing, Suzuka University of Medical Science, Mie, Japan; ^9^Kii ALS/PDC Research Center, Graduate School of Regional Innovation studies, Mie University, Mie, Japan

**Keywords:** amyotrophic lateral sclerosis, parkinsonism-dementia complex, Kii Peninsula, tau, nitrative stress, oxidative stress

## Abstract

**Objective:** The Kii Peninsula of Japan is known to be a high incidence area of amyotrophic lateral sclerosis/parkinsonism-dementia complex (Kii ALS/PDC) with tauopathy. Nitrative stress and oxidative stress on ALS/PDC and their relationship to tau pathology were clarified.

**Methods:** Seven patients with Kii ALS/PDC (3 males and 4 females, average age 70.7 years, 3 with ALS, 2 with ALS with dementia, and 2 with PDC) were analyzed in this study. Five patients with Alzheimer's disease and five normal aged subjects were used as controls. Immunohistochemical analysis was performed on formalin-fixed, paraffin-embedded temporal lobe sections (the hippocampal area including hippocampus, prosubiculum, subiculum, presubiculum, and parahippocampal gyri) using antibodies to detect phosphorylated tau (anti-AT-8), nitrated guanine (anti-8-NG), anti-iNOS, anti-NFκB, and oxidized guanine (anti-8-OHdG) antibodies.

**Results:** Most hippocampal neurons of Kii ALS/PDC patients were stained with anti-8-NG, anti-iNOS, anti-NFκB, and anti-8-OHdG antibodies and some AT-8 positive neurons were co-stained with anti-8-NG antibody. The numbers of 8-NG positive neurons and 8-OHdG positive neurons were greater than AT-8 positive neurons and the number of 8-NG positive neurons was larger in patients with Kii ALS/PDC than in controls.

**Conclusion:** Nitrative and oxidative stress may take priority over tau accumulation and lead to the neurodegeneration in Kii ALS/PDC.

## Introduction

The Kii Peninsula is the high-incidence focus of amyotrophic lateral sclerosis (ALS) and parkinsonism-dementia complex (PDC) in Japan (Kokubo and Kuzuhara, 2001). ALS and PDC occur concomitantly on Guam and in the Kii Peninsula and share common neuropathological features characterized by the presence of many neurofibrillary tangles in the central nervous system (Kokubo and Kuzuhara, 2001, 2004; Kuzuhara, 2004; Kuzuhara and Kokubo, 2005). Kii ALS shows typical ALS symptoms and Kii PDC shows parkinsonism, dementia especially abulia and/or ALS symptoms. Lately Kii ALS/PDC has been revealed multiple proteinopathy including tauopathy, α-synucleinopathy and TDP-43 proteinopathy (Mimuro et al., 2017). Although the pathogenesis of Kii ALS/PDC remains unclear, interaction of environmental factors and genetic factors are surmised to cooperate in the development of the disease.

Oxidative stress (OS) and nitrative stress (NS) are involved in many neurodegenerative diseases, such as Parkinson's disease (PD), Alzheimer's disease (AD), and ALS (Jesberger and Richardson, 1991; de la Monte et al., 2000; Giasson et al., 2002; Kikuchi et al., 2002; Nunomura et al., 2004; Imaizumi et al., 2012). We have reported an increased ration of urinary 8-hydroxydeoxyguanosine (8-OHdG)/creatinine in Kii ALS/PDC patients (Morimoto et al., 2009). In this study, we investigated the interaction between OS, NS, and tau in the pathogenesis of Kii ALS/PDC using immunohistological analyses with the following five antibodies: anti-8-nitroguanine (8-NG), which detects nitrated guanine in DNA and RNA (Ma et al., 2004, 2006; Pinlaor et al., 2004a,b), anti-8-OHdG, which detects oxidized guanine in RNA (Kikuchi et al., 2002; Nunomura et al., 2004), anti-inducible nitric oxide synthase (iNOS) (Levecque et al., 2003; Fernández-Vizarra et al., 2004; Ma et al., 2004, 2006; Pinlaor et al., 2004a,b), anti-NFκB which induces iNOS, and anti-AT-8 antibody, which detects phosphorylated tau (p-tau). We found that OS and especially NS, were concerned with tau deposition in the Kii ALS/PDC patients' brains.

## Materials and methods

### Patient tissue

The brains from seven patients with Kii ALS/PDC, five from patients with AD and five normal aged control subjects were analyzed (Wilcoxon test for age: Control vs. AD; *p* = 0.1172, Control vs. Kii ALS/PDC; *p* = 0.1062). Informed consents were obtained from the families of all patients who participated in the present study in the written form. The present study was approved by the Ethical Committee of Mie University Hospital, Mie, Japan (approval number; 2592) and Tokyo Metropolitan Geriatric Hospital, Tokyo, Japan (approval number; 2014-11, 2014-12). The profiles of these patients are summarized in Table 1. The clinical and neuropathological findings of Kii ALS/PDC have been described in previous reports (Kuzuhara et al., 2001; Mimuro et al., 2017). Neuropathological diagnosis of AD was obtained based on the following criteria: (1) Braak neurofibrillary tangle stage IV or above and (2) amyloid deposits of Braak stage C.

**Table 1:** The quantitative assessment summary of the immunohistochemistry in the hippocampus of Kii ALS/PDC patients, Alzheimer's disease patients and controls.

**Case**	**Range of age at death**	**Clinical phenotype**	**AT-8**	**8-NG**	**iNOS**	**NF-κB**	**8-OHdG**
Kii ALS/PDC-1	61–65	ALS	+	++	++	+	+
Kii ALS/PDC-2	81–85	ALS	++	+++	++	+	+++
Kii ALS/PDC-3	66–70	ALS	++	+++	+++	++	+
Kii ALS/PDC-4	71–75	ALS with D	+++	+++	+++	+++	++
Kii ALS/PDC-5	61–65	ALS with D	++++	+++	+++	++	++
Kii ALS/PDC-6	66–70	PDC	+	+	+	+	−
Kii ALS/PDC-7	71–75	PDC	++	+++	+++	+++	++
AD-1	76–80	D	++	+	++	+	+
AD-2	81–85	D	++	++	++	++	+
AD-3	86–90	D	+++	+	+	+	++
AD-4	86–90	D	+++	++	++	++	++
AD-5	76–80	D	+++	++	++	++	++
Control-1	51–55	−	−	−	−	−	−
Control-2	61–65	−	−	−	−	−	+
Control-3	96–100	−	−	+	+	−	+
Control-4	66–70	−	−	+	+	+	+
Control-5	56–60	−	−	+	+	+	+

*Immunohistochemical grading was performed based on frequency of the staining results, as described previously. The frequency of positive cells in a section was scored as negative (–), less than 25% (+), 26–50% (+ +), 51–75% (+ + +), or more than 76% (+ + + +). AD, Alzheimer's disease; ALS, amyotrophic lateral sclerosis; D, dementia; PDC, Parkinsonism-Dementia Complex; 8-NG, 8-Nitroguanine; 8-OHdG, 8-hydroxy-2′-deoxyguanosine; iNOS, inducible nitric oxide synthase; NF-κB, nuclear factor-kappa B*.

### Immunohistochemical study

For immunohistochemical analyses, paraffin-embedded, 6 μm sections from the hippocampal area including hippocampus, prosubiculum, subiculum, presubiculum, and parahippocampal gyri were cut and incubated with a range of antibodies as follows: rabbit polyclonal anti-8-NG antibody (1:100, provided by Dr. Ma, Suzuka University of Medical Science, Mie, Japan) for specific detection of nitrated guanine in DNA and RNA, the production and properties of which were reported previously (Ma et al., 2004, 2006; Pinlaor et al., 2004a,b), anti-8-OHdG antibody, a mouse monoclonal antibody for detection of oxidized guanine in RNA (1:30; Trevigen, Gaithersburg, MD, USA) (Kikuchi et al., 2002), anti-iNOS antibody for detection of the inducible form of nitric oxide synthase (1:500; Sigma-Aldrich Japan, Tokyo, Japan) (Levecque et al., 2003; Fernández-Vizarra et al., 2004; Ma et al., 2004, 2006; Pinlaor et al., 2004a,b), anti-NF-κB antibody, a mouse monoclonal antibody against the p65 subunit (1:400; Sigma) (Sugiura et al., 2013) and anti-AT-8, a mouse monoclonal anti-phosphorylated tau antibody [1:100; Innogenetics (Fujirebio Europe N.V.), Ghent, Belgium].

Double Immunofluorescence labeling were performed on sections from paraffin-embedded brain samples that had been fixed in 4% paraformaldehyde or in 10% formalin. For immunostaining, following de-paraffinization with xylene, sections were hydrated through graded ethanol concentrations. After washing three times with phosphate buffered saline (PBS), the brain sections were treated with 1% skim milk in PBS for 30 min to block non-specific bindings. Subsequently, section was treated with 8-NG and/or iNOS, NF-κB, 8-OHdG, AT-8 for overnight at room temperature. And then treated with Alexa 594-labeled goat antibody against rabbit IgG and Alexa 488-labeled goat antibody against mouse IgG (1:400 diluted in PBS, Molecular Probes, Eugene, OR, USA) for 3 hours. The nuclei were stained with DAPI (Dapi FluoromountG, Birmingham, AL, USA). The stained sections were examined under a fluorescence microscope (BX53, Olympus, Tokyo, Japan).

According to previous reports (Ma et al., 2011; Thanan et al., 2013), immunohistochemical grading was defined based on frequency derived from the staining results. The frequency of positive cells in a section was scored as negative (−), less than 25% (+), 26–50% (++), 51–75% (+ + +), or more than 76% (++++).

## Results

Seven Kii ALS/PDC patients showed positive staining for AT-8, 8-NG, iNOS, and NF-κB (100%), and six patients showed positive staining for 8-OHdG (86%). Five AD patients also showed positive staining for AT-8, 8-NG, iNOS, NF-κB, and 8-OHdG (100%). Three control subjects showed positive staining for 8-NG and iNOS (60%), two controls showed positive staining for NF-κB (40%), and four controls showed positive staining for 8-OHdG (80%).

The number of neurons positive for AT-8, 8-NG, iNOS, NF-κB, and 8-OHdG in the patients with Kii ALS/PDC or AD was greater than that in the control subjects (Table 1). Additionally, comparing the grouped (−) and (+) against the grouped (+ +), (+ + +), and (+ + + +) in the score for the frequency of positive cells, statistical analysis using two-sided Fisher's exact test showed the following; AT-8 (Control vs. AD: *p* = 0.0079, Control vs. Kii ALS/PDC: *p* = 0.0278), 8-NG (Control vs. AD: *p* = 0.1667, Control vs. Kii ALS/PDC: *p* = 0.0152, iNOS (Control vs. AD: *p* = 0.0476, Control vs. Kii ALS/PDC: *p* = 0.0152), NF-κB (Control vs. AD: *p* = 0.1667, Control vs. Kii ALS/PDC: *p* = 0.0808), and 8-OHdG (Control vs. AD: *p* = 0.1667, Control vs. Kii ALS/PDC: *p* = 0.0808).

**Figure 1:**
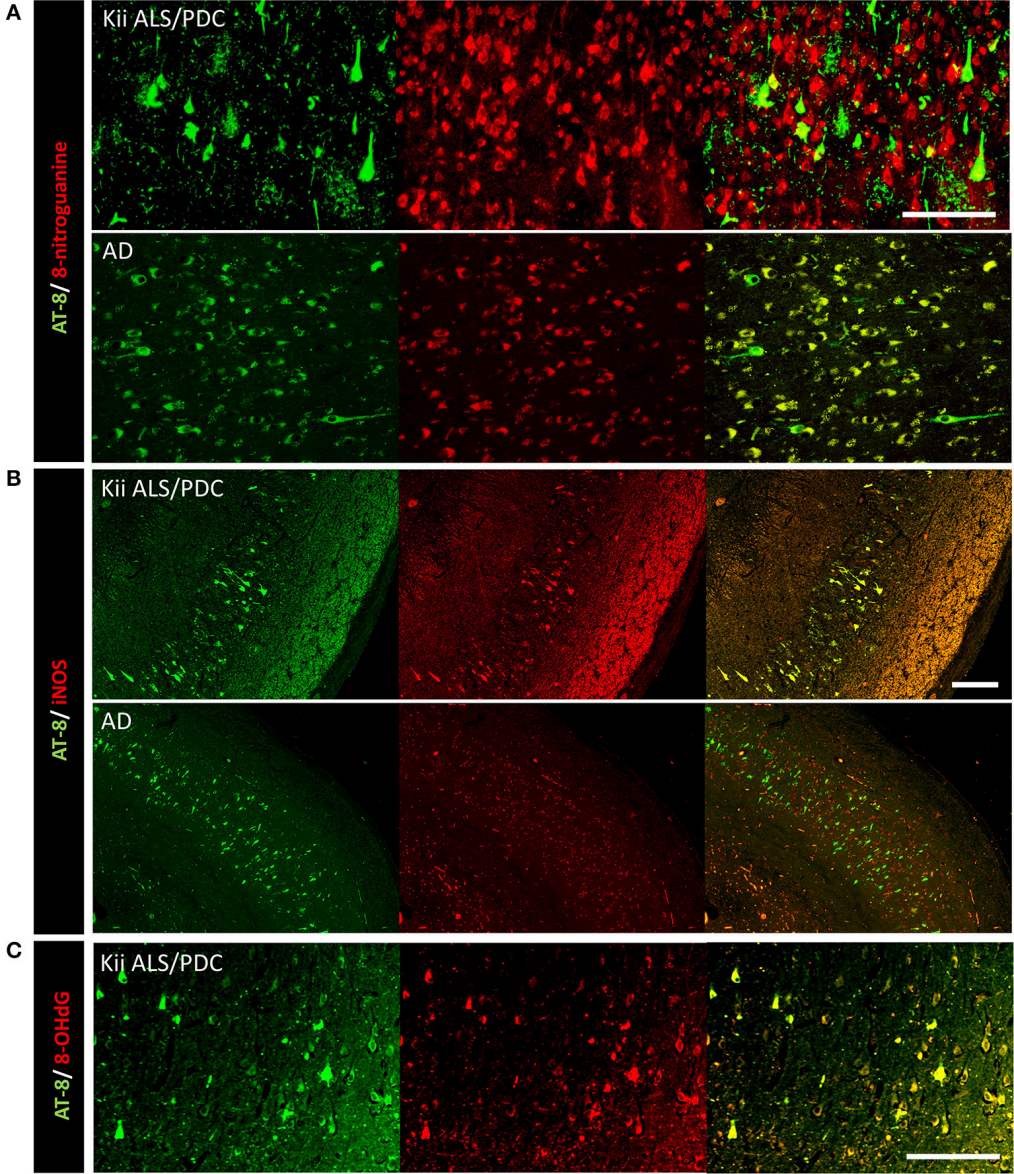
Double immunofluorescence staining in the hippocampus of patients with Kii ALS/PDC and Alzheimer's disease. **(A)** AT-8 and anti-8-NG antibody. **(B)** AT-8 and anti-iNOS antibody. **(C)** AT-8 and anti- 8-OHdG antibody. **(A**: × 200, **B**: × 100, **C**: × 200, scale bars represent 50 μm).

**Figure 2:**
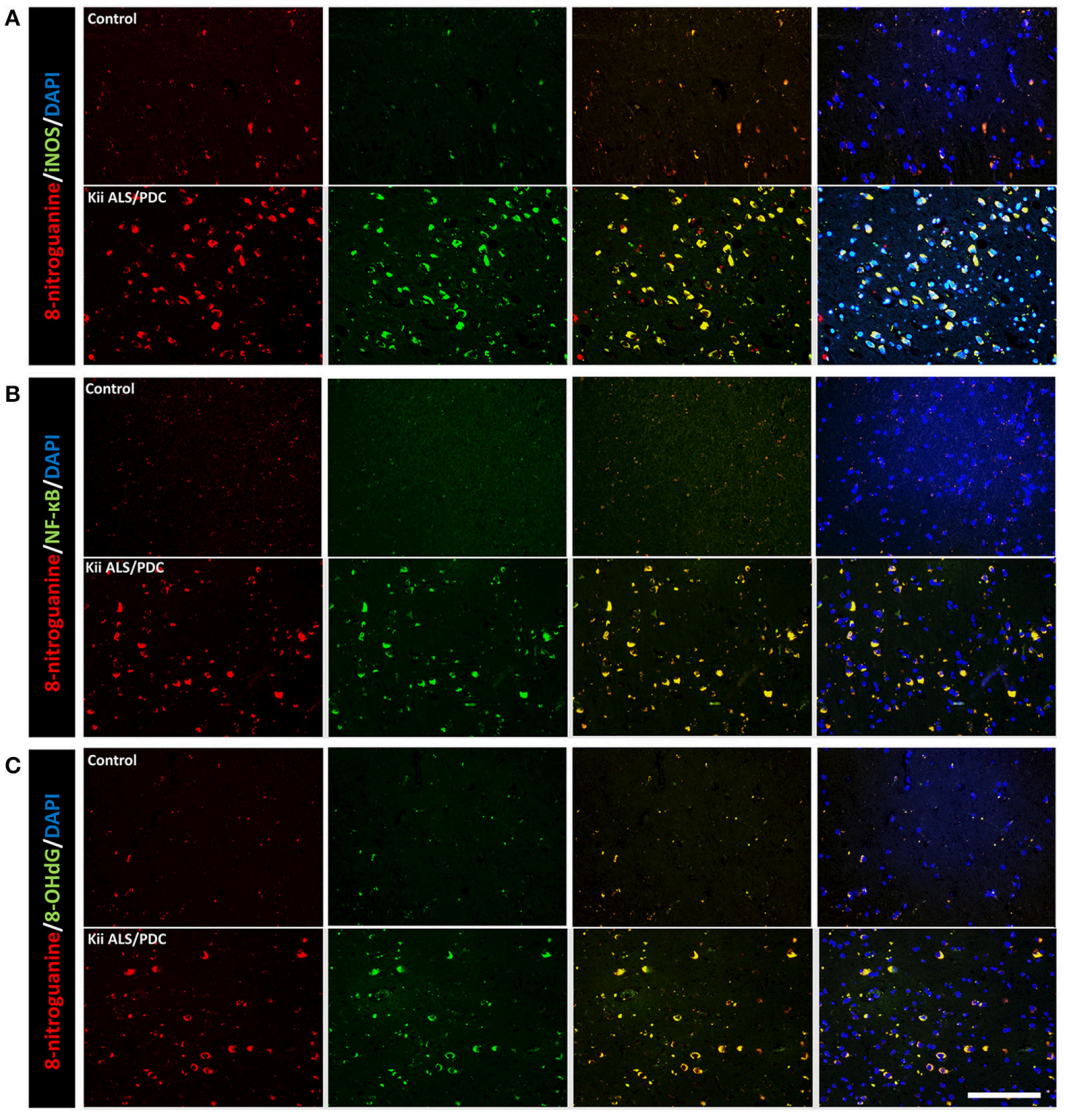
Double immunofluorescence staining in the hippocampus of control and Kii ALS/PDC patients. **(A)** Anti-8-NG antibody and anti-OHdG antibody. **(B)** Anti-8-NG antibody and anti-NF-κB antibody. **(C)** Anti-8-NG antibody and anti- iNOS antibody (all images × 200, scale bar represents 50 μm).

Double immunofluorescence staining of AT-8 and 8-NG showed that some neurons with co-staining of both antibodies in Kii ALS/PDC brains (Figure 1). The number of anti-8-NG antibody positive neurons in each patient was greater than that of AT-8 positive neurons (Figure 1A). Double immunofluorescence staining of AT-8 and 8-OHdG, and AT-8 and iNOS, showed that most of the AT-8 positive neurons were also co-stained with anti-8-OHdG or anti-iNOS antibodies (Figures 1B,C). Double immunofluorescence staining with each pair of antibodies; 8-NG and iNOS, 8-NG and NF-κB, and 8-NG and 8-OHdG, showed co-staining in control and Kii ALS/PDC brains (Figure 2). Overall, the positive expression of 8-NG, iNOS, NF-κB and 8-OHdG was 100, 100, 100, and 86% of cells, respectively, in the hippocampal area including hippocampus, prosubiculum, subiculum, presubiculum, and parahippocampal gyri in the cerebrum of the Kii ALS/PDC patients.

## Discussion

Regarding the pathomechanism of Kii ALS/PDC, environmental factors such as mineral deficiency have been proposed. Yase (1972) advocated that a combination of hypo-calcium and hypo-magnesium in the drinking water induces secondary hypoparathyroidism, leading to mineral deposition and neuronal cell death. Morimoto et al. (2009) revealed an increase in the ratio of urinary 8-OHdG versus creatinine in Kii ALS/PDC patients, while Kihira et al. suggested OS associated with lifestyle changes might be related to the decrease of Kii ALS/PDC (Kihira et al., 2017). Kokubo et al. (2012) reported that the free radical scavenger, Edaravon, improved volition in patients with ALS/PDC.

**Figure 3:**
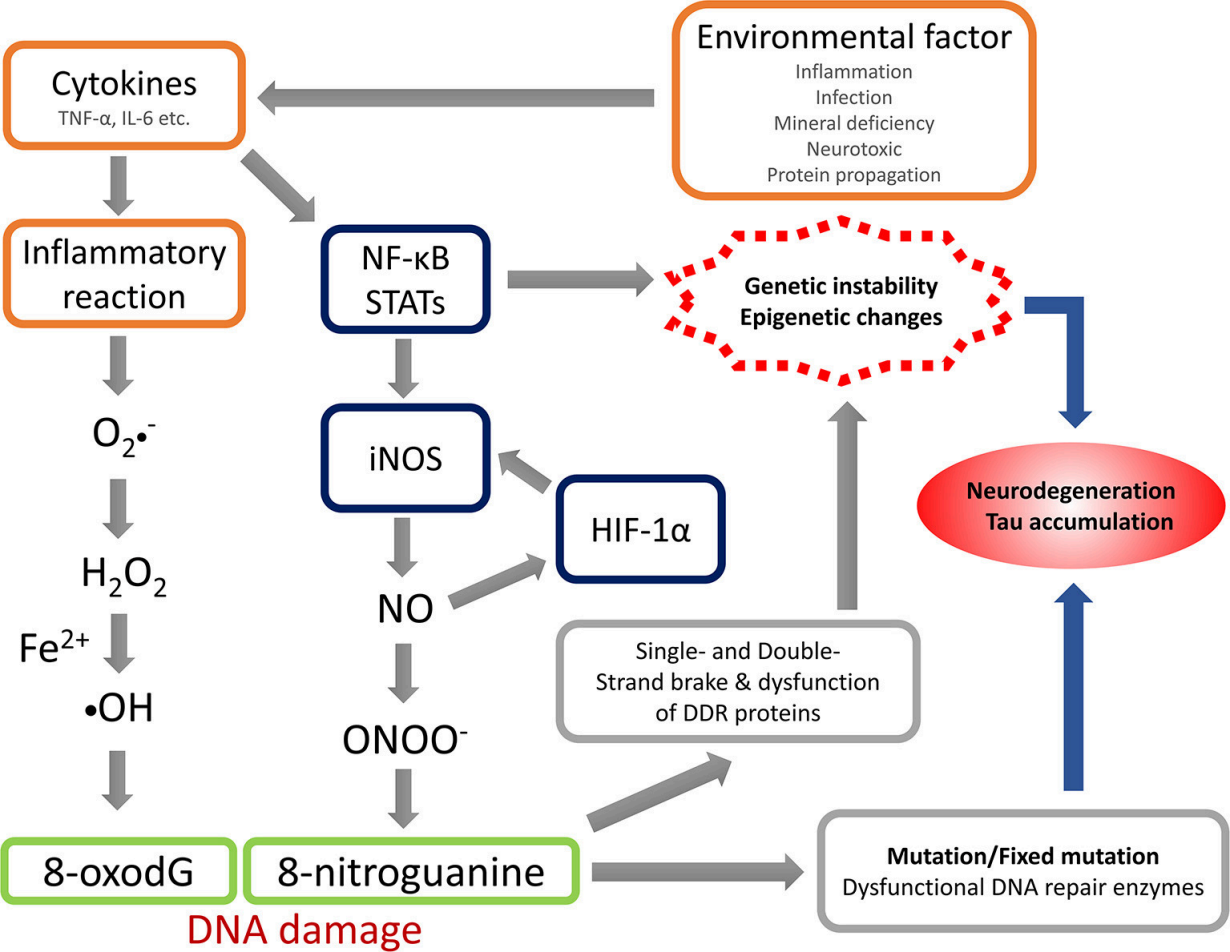
Hypothesis of nitrative stress in the pathomechanism of Kii ALS/PDC. TNF-α, tumor necrosis factor-α; IL-6, interleukin-6; NF-κB, nuclear factor-kappa B; STATs, signal transducers and activators of transcriptions; iNOS, inducible nitric oxide synthase; HIF-1α, hypoxia Inducible Factor-1α; NO, nitric oxide; DDR, DNA damage response.

The anti-8-NG antibody recognizes the nitrated guanine in DNA and RNA, reflecting NS. The present study revealed that OS and NS are highly involved in the pathogenic mechanism of Kii ALS/PDC, and the nitration could be induced by NO generated via iNOS. Under the condition with acceleration of their production or impairment of the normal reduction, OS and NS can be arise and accelerated or when the mechanisms involved in maintaining the normal reductive cellular environment are impaired. The summation of oxidative and nitrative modifications changes the conformation of key proteins, which contributes to neuronal cell death throughout neuronal dysfunction (Giasson et al., 2002; Figure 3). The physiological changes induced by aging may facilitate the accumulation of abnormal proteins through OS and NS (Oliver et al., 1987; Harman, 1992; Stadtman, 1992; Ames et al., 1993; Reiter, 1995; Giasson et al., 2002). While OS and NS has been implicated in many diseases like AD, PD, ALS (Bergeron, 1995; Good et al., 1996; Jenner and Olanow, 1996; Giasson et al., 2002) and Kii ALS/PDC (Morimoto et al., 2009), OS and NS may be promoted by genetic predisposition and/or environmental factors accumulated in the high incidence area or lower the cellular capacity to compensate for such insults.

Nitrative stress and oxidative stress can be exceedingly toxic to neurons. In a state of neurodegeneration, which progress over many years, the aberrant and continuous nitrative and oxidative stress may lead to the accumulation of abnormal proteins and cell death (Giasson et al., 2002). The 8-NG anion radical is formed by NADPH/P450 reductase. Generation of Superoxide (O2^−.^) from NOSs is stimulated by 8-NG and 8-NG prompts O2^−.^ generation by means of reductive activation of NOSs at the reductase domain. The previous study shows that NOS uncoupling, and thereby formation of O2^−.^, might be related to many disorders through the following mechanisms: (1) production of reactive metabolites including hydrogen peroxide, (2) consumption of NO via the rapid reaction with O2^−.^ to form peroxynitrite (ONOO−), and (3) peroxynitrite-induced oxidative and nitrative injuries (Sawa et al., 2003).

Next, we mention the relationship between OS/NS and tau protein. Some studies revealed that chronic OS leaded to increased phosphorylation of tau in culture neurons (Zhu et al., 2005; Su et al., 2010). Furthermore, carbonyl-4-hydroxy-2-nonenal (4-HNE) facilitates aggregation of p-tau *in vitro* (Pérez et al., 2000) and induces hyperphosphorylation of tau (Gómez-Ramos et al., 2003; Liu et al., 2005). In primary cortical neuron cultures of rat treated with cuprizone (a copper chelator in combination with oxidant agents Fe^2+^ and H_2_O_2_), Glycogen synthase kinase-3 beta (GSK-3β) activity and pathologic hyperphosphorylation of tau significantly increased (Lovell et al., 2004; Lee et al., 2007). Particularly, some tau kinases belong to the family of stress-activated protein kinases, which are activated in response to OS (Goedert et al., 1997; Atzori et al., 2001). Interestingly, HNE directly activates two members of the stress-activated kinase family (JNK and p38) in NT2 neuronal cells (Tamagno et al., 2005). Another pathologic link between abnormal phosphorylation of tau and OS is peptidyl prolyl cis-trans isomerase 1 (PPIase1) or Pin1. This enzyme is significantly downregulated and oxidized in hippocampus of AD patients. Because Pin1 relates to de-phosphorylation of tau, *in vivo* oxidative modifications of Pin1 found in hippocampus of AD patients reduce Pin1 activity, leading to increased phosphorylation of tau (Sultana et al., 2006). Additionally, mitochondrial oxidative stress causes hyperphosphorylation of tau (Melov et al., 2007).

On the other hand, there is also substantial evidence for nitrative damage as revealed by immunostaining of NFTs for 3-nitrotyrosine (3-NT) (Good et al., 1996; Smith et al., 1997). Moreover, levels of 3-NT and o-o-dityrosine detected analytically are greatly increased in AD brains (Hensley et al., 1998), which is consistent with the increase in 3-NT cytoplasmic immunoreactivity in many neurons (Smith et al., 1997). Interestingly, astrocytic nitric oxide triggers tau hyperphosphorylation in hippocampal neurons (Saez et al., 2004). Nitrated and Thioflavin-S-positive tau aggregates were produced in a oligodendrocytic cell line treated with peroxynitrite, so this finding implies that nitrative injury is directly linked to the formation of filamentous inclusions of tau (Horiguchi et al., 2003). As another hypothesis, CAPON (carboxy-terminal PDZ ligand of nNOS), a cytoplasmic protein whose C terminus binds to the PDZ domain of nNOS (Jaffrey et al., 1998), may prompt tau phosphorylation and multimerization.

Overall, regarding tau, OS and NS, OS and NS increases oligomerization of truncated tau and hyperphosphorylation of tau which may facilitate tau accumulation in neurons and speed up the process of neurodegeneration (Thanan et al., 2008; Filipcik et al., 2009).

There is other important evidence that the pathogenesis of some neurodegenerative disorders, including AD, PD, and ALS might be involved in the generation of reactive oxygen species (ROS) and/or reactive nitrogen species (RNS), which are related to mitochondrial abnormality. Such defects in respiratory complex activities are possibly related to imbalance of oxidant and antioxidant, and they are said to underlie abnormalities in energy metabolism and induce degeneration of cells (Good et al., 1996; Stewart et al., 2000; Dhir et al., 2008). Mitochondria have multifarious functions and might be essentially significant for adult-onset neurodegenerative disorders including ALS, AD, and PD (Nicholls, 2002). For example, an increased hyperphosphorylation of tau parallels mitochondrial dysfunction and OS in deficient mice in mitochondrial SOD-2 (Melov et al., 2007). As well as cell models and animal models of the neurodegenerative disorders, morphological and biochemical data from analyses of human CNS autopsy, imply that mitochondrial abnormality is a trigger or a propagator of neurodegeneration. New pathomechanisms for mitochondrial disorders and neurodegeneration might be involved in 1-methyl-4-phenyl-1,2,3,6-tetrahydropyridine (mPTP). There is precedence for this logic in mouse models of AD (Du et al., 2008) and ALS (Martin et al., 2009). mPTP involves actively in the pathomechanisms of motor neuronal cell death in ALS mice in a gender-preferential intergenerational pattern (Martin et al., 2009). Therefore, activation of mPTP is a possible trigger for degeneration of motor neurons, and selective vulnerability of motor neurons in ALS might be associated with the trafficking, amount, and composition of mitochondria in the cells.

The results of this study suggest Kii ALS/PDC, other neurodegenerative disorders and aging may have similar mechanisms which associated to the etiology. Furthermore, the increase of OS and NS seen in the Kii ALS/PDC autopsy brain should be reproduced in induced pluripotent stem cells (iPSCs)-derived neurons of Kii ALS/PDC patients as shown in our recent report (Imaizumi et al., 2012), if the Kii ALS/PDC patients have genetic factors underlying the disease development. This is our important future task.

## Conclusion

NS and/or OS may result in different mechanisms of modification of tau protein which influence the stable tau fibrils formation. Given that markers of NS and OS were highly expressed in the brains of Kii ALS/PDC patients, it suggests the involvement of NS and OS in the disease mechanism.

## Author contributions

YH: Research project: Conception and Execution; Manuscript: Writing of the first draft; NM: Research project: Conception and Execution; Manuscript: Writing of the first draft; MY: Research project: Execution; SaM: Research project: Execution; Manuscript: Review and Critique; HO: Research project: Execution; ShM: Research project: Execution; ShoK: Research project: Execution; ShiK: Research project: Conception and Organization; YK: Research project: Conception; Organization and Execution; Manuscript: Review and Critique.

### Conflict of interest statement

The authors declare that the research was conducted in the absence of any commercial or financial relationships that could be construed as a potential conflict of interest. The reviewer JS declared a past co-authorship with one of the authors HO to the handling Editor.
